# A meta-analysis reveals the effectiveness of probiotics and prebiotics against respiratory viral infection

**DOI:** 10.1042/BSR20203638

**Published:** 2021-03-10

**Authors:** Fangyan Wang, Binhui Pan, Sheng Xu, Zhihua Xu, Tiaotiao Zhang, Qihao Zhang, Yifan Bao, Yingwei Wang, Jiamin Zhang, Changlong Xu, Xiangyang Xue

**Affiliations:** 1 Department of Pathophysiology, School of Basic Medicine Science, Wenzhou Medical University, Wenzhou, Zhejiang Province, China; 2 The Basic Research Division of Virology Institute, Wenzhou Medical University, Wenzhou, Zhejiang Province, China; 3 Department of Gastroenterology, The Second Affiliated Hospital and Yuying Children’s Hospital of Wenzhou Medical University, Wenzhou, Zhejiang Province, China; 4 Department of Microbiology and Immunology, School of Basic Medicine Science, Wenzhou Medical University, Wenzhou, Zhejiang Province, China

**Keywords:** Antivirus, Gut microbiota, Gut-lung axis, Prebiotics, Probiotics, Viral pneumonia

## Abstract

Experimental experience suggests that microbial agents including probiotics and prebiotics (representative microbial agents) play a critical role in defending against respiratory virus infection. We aim to systematically examine these agents’ effect on respiratory viral infection and encourage research into clinical applications. An electronic literature search was conducted from published data with a combination of a microbial agents search component containing synonyms for microbial agents-related terms and a customized search component for respiratory virus infection. Hazard ratio (HR), risk ratio (RR) and standard deviation (SD) were employed as effect estimates. In 45 preclinical studies, the mortality rates decreased in the respiratory viral infection models that included prebiotics or prebiotics as interventions (HR: 0.70; 95% confidence interval (CI): 0.56–0.87; *P*=0.002). There was a significant decrease in viral load due to improved gut microbiota (SD: −1.22; 95% CI: −1.50 to −0.94; *P*<0.001). Concentrations of interferon (IFN)-α (SD: 1.05; 95% CI: 0.33–1.77; *P*=0.004), IFN-γ (SD: 0.83; 95% CI: 0.01–1.65; *P*=0.05) and interleukin (IL)-12 (SD: 2.42; 95% CI: 0.32–4.52; *P*=0.02), IL-1β (SD: 0.01; 95% CI: −0.37 to 0.40; *P*=0.94) increased, whereas those of TNF-α (SD: −0.58; 95% CI: −1.59 to 0.43; *P*=0.26) and IL-6 (SD: −0.59; 95% CI: −1.24 to 0.07; *P*=0.08) decreased. Six clinical studies had lower symptom scores (SD: −0.09; 95% CI: −0.44 to 0.26; *P*=0.61) and less incidence of infection (RR: 0.80; 95% CI: 0.64–1.01; *P*=0.06). Our research indicates that probiotics and prebiotics pose a defensive possibility on respiratory viral infection and may encourage the clinical application.

## Background

Annually, approximately 200 million people experience viral community-acquired pneumonia (CAP) worldwide [1], 24.5% of which were infected with respiratory syncytial virus (RSV), influenza virus and rhinovirus among the most prevalent types [2]. According a review in 2015, an estimated 300–500 million severe cases of the CAP are caused by infection with influenza virus, whereas nearly 14000 in-hospital deaths are related to RSV infection [3]. The emergence of the infectious diseases especially severe acute respiratory syndrome (SARS) and Middle East respiratory syndrome (MERS) caused by coronavirus, has led to the life-threatening nature of viral pneumonia.

More recently, novel coronavirus pneumonia (COVID-19) has become a pandemic catastrophe, with a fatality rate rising to 49% in critical cases [4]. At the time of this analysis, updated by the World Health Organization (WHO) for the COVID-19 pandemic, the pandemic caused by the SARS-CoV-2 coronavirus infection had brought over 1.9 million deaths globally out of 88 million [5]. Depending on the host’s immunocompetence, the severity of this respiratory disease can vary from mild symptoms to fatal complications, such as acute respiratory distress and multiple organ dysfunction [6]. However, current strategies to combat SARS-CoV-2 are far from satisfactory. Other than the two neuraminidase inhibitors: acyclovir and ganciclovir, there are few efficacious and efficient antiviral agents.

Preclinical research on the pathology of the viral pneumonia has shown that microbial agents including probiotics (exogenous salutary bacteria like *Bifidobacterium*) and prebiotics (indigestible substance promoting the growth of salutary bacteria, such as oligosaccharides) may modulate the composition of gastrointestinal flora and provide beneficial effects for patients with respiratory diseases, attributing to the gut–lung axis theory. When mice were manually exposed to a germ-free state through antibiotic treatment, both virus-specific CD4 and CD8 T cells and the influenza-specific antibody levels were markedly decreased, leading to a delay of the invasive virus elimination [7]. It was observed that the population of intestinal *Bacteroidetes* was significantly increased while the content of *Firmicutes* was decreased in murine models of RSV. By administering probiotic supplements, the incidence of upper respiratory tract infections was reduced and the disease duration was shortened. According to the microbiota–gut–lung axis theory, the gut microbiota plays the critical role in response to viral lung infections [8].

So far, the studies on probiotics and prebiotics treatments for respiratory viral infections failed to yield the expected results. Thus, in the present study, we proposed and evaluated the existing scientific evidence and credible results from the published preclinical and clinicalstudies, aiming to provide some useful information and suggestions for the future studies of stopping the rampant respiratory virus infection.

## Methods

### Search strategy

We conducted a meta-analysis following the recommended Preferred Reporting Items for Systematic Reviews and Meta-analyses (PRISMA) guidelines. We searched for the published studies up to December 2020 in PubMed, the Cochrane Library, Embase, the China National Knowledge Infrastructure, the Chinese Biomedical Literature Database and VIP databases, using combinations of a microbial agents search component containing synonyms for microbial agents-related terms and a customized search component for viral pneumonia. Retrieved articles were exported to an EndNote file. After removing duplicates, we screened the titles, abstracts and full texts according to the study selection criteria. There was no restriction for language, and a consensus was reached by mutual discussion.

### Study selection

We included only the randomized controlled studies that investigated directly the effects of microbial agents on the respiratory viral infections. We excluded the articles that reported only the preventive effects on respiratory infection events which also contained bacteria-related events but did not specifically refer to the viral infection. We also excluded the comments, letters and other similar articles from which no relevant data could be extracted. There were no restrictions for specific variables such as different virus species and types of microbial agents.

### Quality assessment and data extraction

To assess the quality for the studies included, we applied the Cochrane Risk Assessment Scale to the clinical studies, and used SYRCLE’s risk of bias tool (RoB) for animal studies. The following general information from the studies were extracted: first author, publication year, age, sex, animal species, sample size, condition at baseline, respiratory virus pathogens, intervention (type, dose, duration), control, samples collected for outcome evaluation and effect estimates. Disagreements between the authors during the data abstraction were resolved by referring to the original article.

### Statistical analysis

We evaluated the heterogeneity between study-specific estimates using the *I^2^* statistic and Cochran’s Q test, for which an *I^2^* value >50% or a *P*-value <0.10 was considered as significant heterogeneity. Considering the significant differences in study design among the selected studies, we pooled data using a random-effects model. In cases with significant heterogeneity, we carried out subgroup and sensitivity analyses if permitted. We also estimated the publication bias using Egger’s test. The analyses for preclinical and clinical trials were conducted separately. We extracted data separately for evaluation from various sampling locations, microbial agents types and viral species in the individual studies. We employed the software applications GetData 2.20, RevMan 5.3 and Stata 12.0 for the data extraction and synthesis. For all statistical procedures (except for heterogeneity), we defined a *P*-value of <0.05 as statistically significant.

## Results

### Literature search

**Figure 1:**
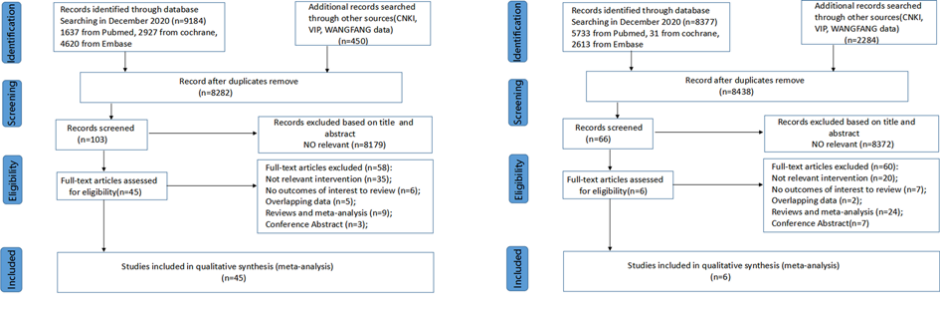
Flow diagram according to the PRISMA protocol We retrieved related articles from databases and included preclinical studies (**A**) and clinical studies (**B**).

As shown in Figure 1A,B, the broad database searches resulted in 9634 preclinical and 10661 clinical hits, which we reduced to 8282 and 8438, respectively, after removing duplicates. After screening separately the titles and abstracts meeting with the protocol eligibility criteria, we included preclinical 104 and clinical 65 articles, respectively. After examining the full text, only 45 preclinical and 6 clinical studies were eligible for the data extraction and final evaluation.

## Study characteristics

### Preclinical studies

Among the 45 selected studies (Table 1), most of them (42) were conducted using mice model; only one using chickens, one using preweaned dairy calves [9] and one using pigs [10–12]. Among the challenge viruses, influenza A had the highest frequency, followed by RSV. In terms of microbial agents, the included animal studies mainly determined the effects of administering prebiotics and probiotics on respiratory viral infection, in which different soluble oligosaccharides were regarded as prebiotics, and live or heat-inactivated lactic acid bacteria were selected as probiotics. The datasets from two studies that used ribavirin (a broad-spectrum antiviral drug) were combined as an additional control group [13,14], distinguishing them from the other studies just employing phosphate saline buffer or saline.

**Table 1:** Study characteristics of included preclinical studies investigating the effect of probiotics and prebiotics on respiratory viral infection

Author, year	Animal model	Total sample size	Virus infection	Intervention of experimental group	Duration	Comparison	Outcome
Iwabuchi, 2011	SPF female BALB/c mice, 4 weeks old	20	Intranasally with 5 × 10^6^ pfu of IFV A/PR/8/34 (H1N1)	Lyophilized BB536 (*Bifidobacterium longum*) at a dose of 2109 colony forming unit/0.2 ml/mouse	2 weeks	Saline	Symptom score↓, body weight loss↓, virus titer↓, IFN-γ↓, IL-6↓, IL-10↑
Kim, 2018	Male BALB/c mice, 8 weeks old	24	A/Puerto Rico/8/34 H1N1 (PR8) strain of IAV	Heat-treated *Lactobacillus plantarum* nF1-fortified yogurt 200 μl each day	21 days	PBS solution	Spleen index↑, thymus index↑, IFN-α↓, IL-2↑, IL-12↑, Klrb1, CD69, NK cell cytotoxicity↑, IL-1β mRNA↑, IL-6 mRNA↑, survival rate
Kiso, 2013	Female BALB/c mice, 6 weeks old	145	Influenza A virus (A/California/04/2009 (CA04) virus)	Heat-killed *Lactobacillus pentosu*s b240 a dose of 10 mg/mouse	5 weeks	Saline	Survival rate↑, body weight↑, IL-10↑, GM-CSF, IL-5↑, G-CSF
Kobayashi, 2011	Female BALB/c/Cr Slc (SPF) mice, 5 weeks old	143	IFV A/PR8/34 H1N1 at a dose of 50 μl of 107.5 TCID_50_/ml	Heat-killed *Lactobacillus pentosus* strain b240 (ONRIC b0240: b240) at doses of 0.4, 2 or 10 mg/mouse	3 weeks	Saline	Survival rate↑, virus titer↓, anti-IFV IgA↑, anti-IFV IgG↑, CTLL-2
Chiba, 2013	Female BALB/c mice, 3 weeks old	NM	10^6^ PFU of human RSV strain A2	*Lactobacillus rhamnosus CRL1505* at a dose of 10^8^ cells/mouse/day	5 days	No treatment	Body weight↑, virus titer↓, lung wet:dry weight ratio↑, BAL albumin↓, BAL LDH↓, IFN-β↑, TNF-α, IFN-γ↑, IL-6 ↑, IL-10↑
Maede, 2009	Female C57BL/6 mice, 7 weeks old	45	Intranasally with influenza virus A/FM/1/47(H1N1) 100 pfu/mouse	Heat-killed *Lactobacillus plantarum L-137* (HK-LP) at a dose of 75 mg/kg/day	2 weeks	PBS solution	Survival rate↑, virus titer↓, IFN-β
Mahooti, 2019	Female BALB/c mice, 6–8 weeks old	20	1 LD90 of human influenza virus A/PR/8/34 (H1N1)	*Bifidobacterium bifidum*	21 days	PBS	Stimulation index↑, IgG↑, IgG1↑, IgG2a↑, IFN-γ↑, IL-4↑, IL-6↓, survival rate↑
Park, 2018	Female BALB/c mice, 5 weeks old	42	Intranasally with influenza A (H1N1 and H3N2 subtypes), influenza B (Yamagata lineage) viruses	Heat-killed *Lactobacillus plantarum* (nF1) 0.05 or 10 mg	4 weeks	PBS	Body weight↑, survival rate↑, virus titer↓
Yasui, 2004	BALB/c mice at 2-day-old (male and female) and 2-, 3-, 5-, 7- and 13-week-old (female)	88	Influenza A/PR/8/34 (PR8, H1N1) virus	*Lactobacillus casei* strain *Shirota* of 10⁁8.6 CFU/mouse	3 weeks	Saline	Survival rate↑, virus titer↓, pulmonary NK cells↑, IL-12↑
Youn, 2012	SPF female BALB/c mice, 6 weeks old	30	Influenza A/NWS/33 (H1N1) virus	Live *Lactobacillus rhamnosus* or dead *Lactobacillus rhamnosus*	21 days	Skim milk	Survival rate↑, virus titer↓, IgA ↓, TNF-α↓, IL-6↓, IL-1β↓
Muramatsu, 2012	Male C57BL/6N mice, 8-week-old	21	A/Puerto Rico/8/34 (PR8; H1N1) strain of influenza A virus	β-Glucan derived from *Aureobasidium pullulans*, 2 mg/ml, 0.2 ml/mouse	24 days	PBS	Survival rate↑, body weight loss↓, virus titer↓, IL-1β↓, IFN-γ, TNF-α↓, IL-6 mRNA expression, lung immune cells, CSF2, CSF3, RIG-1, MDA5 mRNA expression
Chen, 2019	Male BALB/c mice, 6 weeks	24	High pathogenicity influenza virus H1N1 (A/FM/1/47)	*Houttuynia cordata polysaccharide* (HCP), 40 mg/kg/day	4 days	Ribavirin (positive control) No treatment (negative control)	Mucosubstances in goblet cells↓, HIH-1α↓, zo-1↑, TLR2 and TLR4 levels↓, IL-1β↓, IL-10↑
Zhu, 2018	Male BALB/c mice, 4–6 weeks old	60	Intranasally with high pathogenicity influenza virus H1N1 A/FM/1/47 at a dose of LD100	20 or 40 mg/kg/day of *Houttuynia cordata* (HCP) once daily	7 days	Ribavirin (positive control) 5‰ CMC (negative control)	Survival rate↑, lung index↓, IgA↑ TLR4, NF-κB, TNF-α↓, IL-6↓, IFN-α↓, IL-1↓, MIP-1↓, RANTES↓, MCP-1↓, IP-10↓
Ohta, 2007	Female BALB/c mice, 5 weeks old	32	Intranasally with Influenza A virus (NWS strain,H1N1) of 2 × 10^5^ PFU/mouse	Cordyceps militaris dissolved in H_2_O (2.5 mg/0.1 ml in PBS) or APS at a dose of 0.1 mg/15 µl per mouse	10 days	H_2_O	Survival rate↑, body weight↑, virus titer↓, TNF-α↑, IFN-γ↑, NO production↑, iNOS expression↑, IL-1β↑, IL-6↑, IL-10↑
Kawashima, 2011	Female BALB/c mice	50	Intranasal with influenza A virus (A/NWS/33,H1N1) (IFV) of 2 × 10^4^ PFU/mouse	*Lactobacillus plantarum strain YU*: LpYU (0.011, 0.21 or 2.1 mg/day)	14 days	Saline	Virus titer↓, IL-12↑, IgE levels↓, IFN-γ↑, IL-4, IL-10, fecal IgA↑, NK cell activity↑, neutralization antibody titer↑
Kondoh, 2012	Male C57BL/6N mice, 6 weeks old	38	Intranasally with A/Puerto Rico/8/34 (H1N1; PR8), 10^3^ PFU of PR8 at 0.05 ml	Heat-treated FK-23(SLFK), 15 mg per mouse	7 days	Saline	Survival rate↑, body weight↑, IL-10↑, IL-1β, TNF-α, IFN-β, IFN-γ mRNA expression
Maruo, 2012	Female BALB/c mice, 8 weeks old	56	Influenza virus A New Caledonia 2099 (H1N1), 25 μl (200 FFU per mouse) intranasally	100 μl of milk fermented with *Lactococcus lactis subsp. Cremoris FC*	12 days	Sterile physiologic saline	Survival rate↑, body weight loss↓, virus titer↓
Hori, 2002	Female BALB/c mice, 15 months old	49	Intranasally with influenza A/PR/8/34 (H1N1) (PR8) virus	*Lactobacillus casei* strain Shirota	4 months	MM-3 diet	NK cell activity↑, viral titer↓, IFN-γ↑, TNF-α↑, IL-4
Jounai, 2015	Female wildtype DBA/2JJcl mice, 6-10 weeks old	37	Intranasal administration of a 25 μl drop containing 64 hemagglutination units (HAU) of mPIV1	Fed AIN93G containing of 1 mg heat-killed *Lactococcus lactis subsp. Lactis* JCM5805/day/mouse and water *ad libitum*	29 days	AIN93G (Oriental Yeast, Tokyo, Japan)	Survival rate↑, body weight loss↓, lung tissue damage↓, expression of MHC class II on pDCs, IFN-α, IFN-β mRNA expression, lung lymphocytes↑
Jung, 2004	Colostrum-deprived 5-day-old pigs	40	Intranasally with 3 ml of tissue culture fluid containing 2 × 10^6^ tissue culture infective doses 50% (TCID_50_)/ml of Swine influenza virus H1N1, SNUVR030925 strain	*Saccharomyces cerevisiae* β-glucan (50 mg/day/pig)	3 days	Culture medium	The severity of clinical sings↓, rectal temperature↓, microscopics lesions↓, nucleic acid↓, IFN-γ↑, NO↑
Takeda, 2011	Female SPF BALB/c mice, 6 weeks old	94	IFV A/PR/8/34 (H1N1), intranasally infected or mock-infected with 500 PFU in 20 μl of PBS	Lactic acid bacteria, *L. plantarum 06CC2* strain, 0.2 ml per mouse twice daily	9 days	Distilled water	Survival rate↑, body weight loss↓, viral titer↓, IFN-γ, IL-12, IFN-γ↑, TNF-α, IL-6↓, infiltrated cells in BALF↓, NK cell activity↑
Bae, 2018	Female BALB/c mice, 5 weeks old	228	A/Korea/01/2009 (2009 pandemic influenza A H1N1 virus, rK09); A/Puerto Rico/8/1934 expressing green fluorescent protein (rPR8-GFP); A/Anhui/01/2013 (avian influenza A H7N9 subtype 6:2 vaccine virus, rAH01)	*Lactobacillus plantarum* (Lp) or *Leuconostoc mesenteroides* (Lm) strain (10^9^ CFU in 200 µl PBS) once daily	14 days	PBS	Survival rate↑, body weight↑, viral plaques↓
Antunes, 2019	Female BALB/c mice, 6–8 weeks old	NM	Intranasally with 10^7^ PFU/ml of RSV A2 strain	A high-fiber diet (HF) containing cellulose and pectin from citrus	4 weeks	Cellulose	Body weight↑, viral load↓, total cells, lung histological score↓, CD11c, CD86 cells in axillary lymph nodes, TNF-α, IL-10↑, IL-4, CD4 T cell↓, IFN-γ, IL-17a, FoxP3-CD11CD86
Belkacem, 2017	Female BALB/c mice, 6 weeks old	NM	Influenza A virus, A/Scotland/ 20/74 (H3N2); IAV 50 μl	*Lactobacillus paracasei CNCM I-1518* strain, 200 μl with 2 × 10^8^ CFU	17 days	PBS	Viral load↓, body weight↑, temperature loss↓, clinical score↑, total cells in lung, gut microbiota, IL-1α, IL-1β↑, MIP-1α, MIP-1β, IFN-γ, MCP-1 IL-33↑, IL-10↑
Chen, 2016	C57BL/6 male mice	NM	Influenza A virus strain (A/WSN/33)	Heat-killed *Enterococcus faecalis*, 17 mg/kg/day	12 days	PBS	Survival rate↑, body weight loss↓, viral titer↓, leukocytes↓, IL-6, TNF-α, IL-1β, IFN-γ, IL-10, IL-17, MCP-1↑, TGFβ
Eguchi, 2019	Female BALB/c mice, 5–7 weeks old	25	RSV A2 strain, 5 × 10^6^ TCID_50_/head	*Lactobacillus gasseri SBT2055*, 2 × 10^9^ cfu/head	25 days	25% trehalose solution	Body weight loss↓, viral titer↓, TNF-α↓, CCL2↓, IL-1β, IL-6↓
Goto, 2013	Female BALB/c mice, 4 weeks old	104	Intranasally with Influenza A/PR/8/34 (H1N1), 5 × 10^5^ plaque-forming units (PFUs)/mouse	Expt I: doses of 300 ml of non-live L-92 suspension/day, Expt II: doses of 300 ml of live L-92 suspension/day	21 days	Saline	Symptom score↓, viral titer↓, NK cell activity↑, consolidation score↓, neutrophils↓, Eotaxin↑, M-CSF↑, IL-1β↑, RANTES↑,IFN-α↑, IL-4, IL-6, IL-17
Jiang, 2017	SPF BALB/c mice, 6–7 weeks old	44	Intranaslly with H9N2 subtype Avian influenza virus at the dose of 0.05 ml 10× LD_50_ of H9N2	Recombinant NC8 strains at the dose of 1.0 × 10^9^ CFU/0.1 mL phosphate buffered saline (PBS)	3 days	PBS	The activation and polarization of T cells, IgA↑, IFN-γ, IL-4, IL-17. survival rate↑, body weight↑
Kawahara, 2015	Female BALB/c mice, 6 weeks old	26	Intranaslly with influenza virus A/Puerto Rico/8/1934 (PR8, H1N1) virus	200 μl of phosphate buffered saline (PBS) containing 2.0 × 10^9^ CFU of *Bifidobacterium longum* MM-2(MM-2)	17 days	PBS	Survival rate↑, symptom score↑, body weight↑, viral titer↓, NK cell activity↑, IFN-α↑, IL-6↓, TNF-α↓
Nakayama, 2014	Male C57BL/6N mice, 5–7 weeks old	34	Intranasally with A/Puerto Rico/8/34 (PR8; H1N1) at a titer of 1000 pfu	*Lactobacillus gasseri SBT2055*	41 days	25% trehalose solution	Survival rate↑, body weight, virus titers, IL-6↓, BALF cells, LDH activity↓, antiviral genes
Nogusa, 2009	Male C57BL/6– mice, 6–8 weeks old	48	Intranasally with influenza A/Puerto Rico/8/34	Active hexose correlated compound at daily doses of 0.05, 0.1, 0.5 and 1 g/kg	17 days	No treatment	Survival rate↑, body weight↑,NK cell cytotoxicity and lytic efficiency↑
Kawase, 2010	Female BALB/c mice, 4 weeks old	39	Intranasally with Flu A/PR/8/34 (H1N1), 5 × 10^6^ PFU per mouse	10 mg of *Lyophilized Lactobacillus rhamnosus GG* and *Lactobacillus gasseri TMC0356* in 200 ul of sterile physiologic saline	19 days	sterile physiologic saline	Clinical symptom score↓, virus titers↓, histopathology
Kechaou, 2013	SPF BALB/c mice, 6 weeks old	49	Intranasally with influenza virus H1N1 strain A Puerto Rico/8/1934 (A/PR8/34; a mouse-adapted strain)	1 × 10^9^ CFU of *Lactobacillus casei DN114-001, Lactobacillus rhamnosus GG* or *Lactobacillus plantarum CNRZ1997*	24 days	PBS	Weight loss↓, clinical symptoms↓, virus proliferation↓
Nagai, 2011	Female BALB/c mice, 7 weeks old	27	Intranaslly with influenza virus A/PR/8/34 (A/PR8, H1N1)	Expt I: yogurt at a dose of 0.4 ml (20 μg as polysaccharide)/mouse/day Expt II: EPS at a dose of 20 μg/mouse(∼1 mg/kg body weight)	27 days	Water	Survival rate↑, virus titers↓,virus antibodies titers↑, NK cell activity↑
Kawase, 2011	Female BALB/c mice, 4 weeks old	48	Intranasally with Flu A/PR/8/34 (H1N1), 5 × 10^6^ PFU per mouse	Heat-killed *Lactobacillus gasseri TMC0356* 10 mg of lyophilized lactobacilli (TMC0356-70, group 1; TMC0356-90, group 2)	19 days	Sterile physiologic saline	Body weight↑, clinical symptom score↑, virus titer↓, histopathology,NK cell activity, (IL)-12↑, IL-15↑, IL-21↑, IFN-c↑, TNF↑, IL-12a↑, IL-12rbl↑, IL-2rb↑, perforin 1↑
Song,	SPF female BALB/c mice, 4 weeks old	20	Intranasally with influenza virus A/NWS/3 3 (H1N1)	0.3 ml of 1 × 10^9^ colony forming units/ml *Lactobacillus rhamnosus M21* (KCTC 10965BP)	34 days	Skim milk	Survival rate↑, pneumonia↓, IL1β, IL-6, IL-4, IFN-γ↑, IL-2↑, IgA levels↑
Park, 2013	Female BALB/c mice, 6–8 weeks old	27	Influenza virus strains A/Puerto Rico/8/1934 (H1N1; A/PR8) and A/Philippines/82 (H3N2 subtype)	Intragastric 200 ml of suspension containing 10^9^ or 10^8^ CFU of live *L. plantarum DK119* once daily	24 days	No treatment	Body weight↑, viral titers↓,IL-4↑, IL-6↑, TNF-a↑, IL-12↑, IFN-γ↑, lung histopathology
Pugh, 2015	Female BALB/c mice, 6–8 weeks old	60	Intranasally with human influenza A/PR/8/34 (H1N1) virus of 5 μl of viral inoculum per nostril	Fed rodent diet AIN-93M supplemented with 0.35% Immulina (the average daily feed intake was 3.0–3.5 g/mouse)	51 days	Rodent diet AIN-93M	Feed intake↑, weight loss↓, clinical signs↓, lung histopathology scores↓,
Seo, 2012	SPF chickens, 5 weeks old	100	Orally challenged with low-pathogenic avian influenza (LPAI) (H9N2)	Live *Leuconostoc mesenteroides YML003* or heat-killed *Leuconostoc mesenteroides YML003*	2 weeks	Normal diet	Antiviral activity↑, viral gene titers↓, IFN-γ levels↑
Waki, 2013	Female SPF BALB/c mice, 7–8 weeks old	60	Intranasally with 2× MLD_50_ (50% mouse lethal dose) IFV A/PR/8/34 (H1N1)	*Lactobacillus brevis KB290 (KB290)*	14 days	Potato starch	Body weight↑, physical conditions↑, IgA level↑, IFN-α↑,
Zheng, 2006	ICR mice of either sex	NM	Intranasally with influenza virus (H1N1)	Exopolysaccharide from *Aphanothece halophytica* (Chroococcales) at doses of 10, 20, 40, 60 and 80 mg/kg/day	4 days	Ribavirin (Sigma) at a dose of 40 mg/kg (positive control), Distilled water (negative control)	Endotoxin level, pulmonary edema↓, NK cells↑, lymphocyte proliferation↑, IL-2↑, IL-1β↓, phagocytic capacity↓
Yin, 2013	Female BALB/c mice, 5 weeks old	28	Intranasal with 50 μl of Influenza A/PR/8/34 virus (H1N1 subtype)	Korean red ginseng (RG) total extract, RG polysaccharide and RG saponin were directly given into digestive tracts of mice by oral feedings	28 days	PBS (negative control), oseltamivir (positive control)	Survival rate↑,body weight↑, TNF-α/inducible nitric oxide synthase (iNOS)-producing dendritic cells (tipDCs)↓
Yitbarek, 2018	1-day-old SPF layer chickens	100	via the oral-nasal route with 400 μl of 10^7^ tissue culture infectious dose 50 (TCID_50_/ml) of A/turkey/Wisconsin/1/ (H9N2) infuenza virus	Administration of probiotic combination (1 ml/day by gavaging to the crop using a 1 ml syringe) or FMT	4 days	Normal diet	Gut microbiota dicersity↓,virus shedding, INF I, IL-22↑, villus, VH:CD↓, Pearson’s correlations
Mahmoud 2020	Holstein×Angus mixed sex	24	NutriTek was fed at a rate of 5 g/day, top-dressed on to the calf starter	SCFP supplemented	31 days	Base milk replacer and calf starter	Body weight↑, clinical score, cortisal, lung pathology, Virus isolation, NS2 copy number, CD4 T↑, CD8 T, δT, IL-6↑, TNFα↑, IL-1β
Trompette, 2018	Female BALB/c or C57BL/6 mice, 8–12 week-old	36	Influenza virus strain PR8 (A/Puerto Rico8/34, H1N1) 1100 PFU for high-dose infection of BALB/c, 4500 PFU for high-dose infection in C57BL/6, and 100 PFU for low-dose infections	Inulin (high-fiber diet)	Since born	cellulose	Survival rate↑,clinical score↓, compliance in response to methacholine↑, RBC accumulation↓, albumin levels↓, MPO activity↓,gut microbial composition, SCFA↑, CXCL1 Production↓, macrophage population↑, CD8^+^ T Cell

Abbreviations: CFU, colony-forming unit; FMT, fecal microbiota transplantation; IAV, influenza A virus; IFN, interferon; IFV, influenza virus; IL, interleukin; NM, not mentioned; PBS, phosphate buffer saline; PFU, plaque forming unit; SCFA, short-chain fatty acid; SPF, specific-pathogen-free.

↑, the effect in intervention group was greater than control group; ↓, the effect in intervention group was smaller than control group.

### Clinical studies

Only six clinical studies were eligible under the predetermined criteria (Table 2), and all were the randomized, double-blind, placebo-controlled trials. Rhinovirus was chosen as the final infectious target in the studies, except for two studies choosing human influenza virus [15] and SARS-CoV-2 [16]. Various probiotics or prebiotics were used. Turner et al. selected *Bifidobacterium animalis* subspecies lactis Bl-04 (Bl-04) as the intervention, while Tapiovaara et al. employed heat-inactivated *Lactobacillus rhamnosus* GG [17,18]. Similarly, Kumpu et al. investigated whether live or heat-inactivated *L. rhamnosus* GG had equivalent effects against rhinovirus, while Tetsu et al. examined the protective effect of *Lactococcus lactis ssp.* lactis JCM5805 [15,19]. Luoto et al. used a mixture of two prebiotics (galacto-oligosaccharide and polydextrose at a 1:1 ratio) and also a probiotic (*L. rhamnosus* GG) as the intervention factors in their study. They wanted to determine the prophylactic effect in 94 preterm infants with the gestational ages greater than 32 + 0 or less than 36 + 6 weeks and birth weight >1500 g [20]. In study from Ettorre et al., participants were 70 hospitalized patients positive for COVID-19. Oral bacteriotherapy using a multistrain formulation, which contained various probiotics such as *Streptococcus thermophilus* and *L. acidophilus*, were selected as intervention.

**Table 2:** Study characteristics of included clinical studies

Author, year	Study design	Sample size	Characteristics of participants	Virus	Intervention	Comparator	Quality assessment	Outcome
Turner, 2017	Randomized, double-blind, placebo controlled, parallel trial	115	Healthy adult volunteers serum neutralizing antibody titer of ≤1:4	Rhinovirus 100 tissue culture infectious dose 50 (TCID_50_) of virus by intranasal drops	*Bifidobacterium animalis subsp. lactis* Bl-04 (Bl-04) a minimum of 2 × 10^9^ cfu of Bl-04 mixed with 1 g of sucrose for 33 days	1 g of sucrose	6	CXCL8↓, CXCL10, G-CSF concentration, virus titer↓, NO, total symptom score
Tapiovaara, 2016	Randomized, double-blind, placebo-controlled, pilot study	50	Healthy subjects aged 18–65 years	Human rhinovirus (HRV)39 100–300 tissue culture infectious dose (TCID)50	Juice enriched with live or heat-inactivated *L. rhamnosus* GG for 6 weeks	Control juice	4	Virus titer↓, NO, total symptom score↓
Luoto, 2014	Randomized, double-blind, placebo-controlled trial	68	Preterm infants gestational age between 32 + 0 and 36 + 6 weeks birth weight greater than 1500 g	Rhinovirus	Prebiotics (galacto_x0002_oligosaccharide and polydextrose mixture, 1:1) probiotic (*L. rhamnosus* GG, ATCC 53103), between days 3 and 60 of life	Microcrystalline cellulose	6	The incidence of RTIs↓, rhinovirus infections↓, virus load↓,
Ettorre, 2020	Randomized, double blind, placebo-controlled, pilot trial	59	Healthy volunteers aged 18–65 years	Rhinovirus immunotype 39 100–300 tissue culture infectious dose (TCID)50	Live or heat-inactivated *L. rhamnosus* GG for 6 weeks	Carrier juice	4	Diarrhea and other symptoms↓, estimated risk of developing respiratory failure↓, prevalence of patients transferred to ICU and mortality↓
Kumpu, 2015	Randomized, placebo-controlled, double-blind, experiment	213	Healthy adults (30–59 years old) from Japan female:male = 121:92	Human influenza virus A/H1N1 (A/PR/8/34)	*Lactococcus lactis ssp. lactis* JCM5805 for 10 weeks	Placebo beverage	5	Occurrence and severity of cold symptoms↓, number of subjects with rhinovirus infection↓
Tetsu, 2015	Randomized, placebo-controlled, double-blind, experiment	213	Healthy adults (30–59 years old) from Japan female:male = 121:92	Human influenza virus A/H1N1 (A/PR/8/34)	*Lactococcus lactis ssp. lactis* JCM5805 for 10 weeks	Placebo beverage	5	Major symptoms of an influenza-like illness↓, IFN-α↑, IGSF15↑

Quality assessment according to the Cochrane Risk Assessment Scale.

Abbreviations: IFN, interferon; TCID, tissue culture infective dose.

↑, the effect in intervention group was greater than control group; ↓, the effect in intervention group was smaller than control group.

### Primary outcomes in preclinical studies

#### Survival analysis

**Figure 2:**
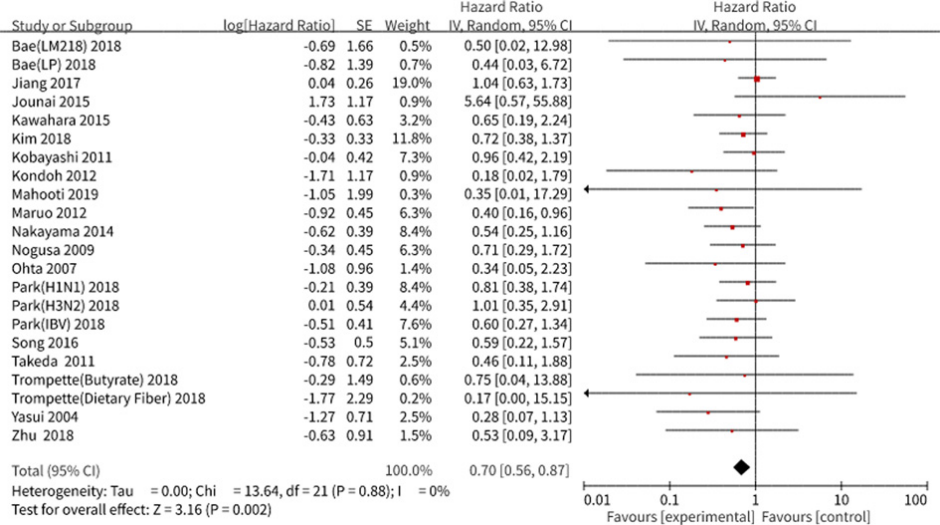
Survival analysis in preclinical studies We performed a forest plot of the survival analysis in preclinical studies using RevMan 5.3. The pooled results are expressed as HRs with their 95% CI. *I^2^* and *P*-values represent the heterogeneity among the studies, while an *I^2^* value >50% and a *P*-value <0.1 indicate considerable heterogeneity. The 95% CI of the result intersecting with the solid vertical line represents no statistical significance. Abbreviations: H1N1, infected with influenza A (H1N1 subtypes); H3N2, infected with influenza A (H3N2 subtypes); IBV, infected with influenza B (Yamagata lineage) viruses; LM218, treated with *Leuconostoc mesenteroides* strain; LP, treated with *Lactobacillus plantarum* 920 strain.

Twenty studies comprising 24 subgroups were included in the meta-analysis using the random effects model. As a result, a pooled hazard ratio (HR) of 0.70, with a 95% confidence interval (CI) of 0.56–0.87 was obtained, which corresponded to a higher survival rate for the microbial intervention group than that for the control group after the virus challenge, without heterogeneity (*I^2^* = 0.0%, *P*=0.88) (Figure 2). Notably, none of the pooled effect estimate in each selected study had statistical significance, probably due to the small sample size except for the data from one study by Maruo et al. [21]. We also evaluated the publication bias using Stata software 12.0, with no quantitative publication bias exists in Egger’s test (*P*=0.10).

#### Viral load

**Figure 3:**
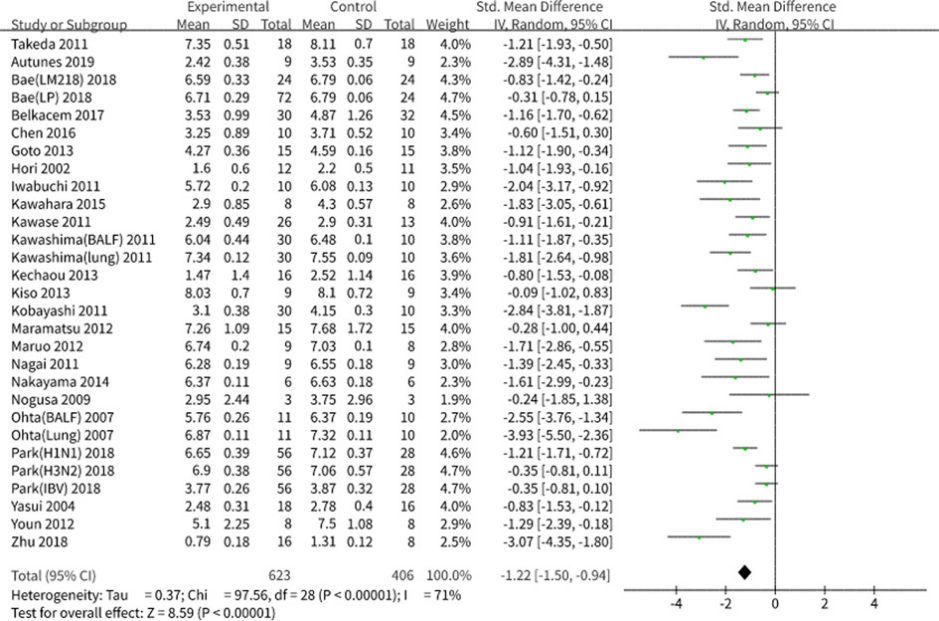
Analysis of viral load in preclinical studies We performed a forest plot of viral load in preclinical studies using RevMan 5.3. We included studies using SD and SEM, transforming SEM to SD for better construction. The data were pooled using a random effects model and expressed as SD with 95% CIs. *I^2^* and *P*-values represent the heterogeneity among the studies, while an *I^2^* value >50% and a *P*-value <0.1 indicate considerable heterogeneity. Abbreviations: LM218, treated with *Leuconostoc mesenteroides* 218 strain; LP, treated with *L. plantarum* 920 strain; IBV, infected with influenza B (Yamagata lineage) viruses; H3N2, infected with influenza A (H3N2 subtypes); H1N1, infected with influenza A (H1N1 subtypes); Heat-killed, treated with heat-killed *Enterococcus faecalis*; Lung, virus titers detected in the lung; BALF, virus titers detected in bronchoalveolar lavage fluids.

Results showed a pooled standard deviation (SD) of −1.22 and 95% CI of −1.50 to −0.94 (*P*<0.001), revealing that the consumption of probiotics or prebiotics alleviated the viral load after a respiratory viral infection (Figure 3). Due to the significant heterogeneity (*I^2^* = 71.3%, *P*<0.001), we performed a subgroup analysis and sensitivity analysis (Table 3). We observed that the heterogeneity would be significantly affected if the eligible studies were grouped into ‘SD’ (*I^2^* = 79%, *P*<0.001) and ‘standard error of the mean (SEM)’ (*I^2^* = 22.3%, *P*=0.24) based on the effect estimates adopted by the individual authors. We sequentially analyzed the studies in which ‘SD’ was employed as the effect estimate. Heterogeneity was significantly affected when the studies were grouped into ‘H1N1’ (*I^2^* = 73%, *P*<0.001) and ‘others’ (*I^2^* = 0.0%, *P*=0.53) according to the specific virus challenge but was not affected when the studies were divided into ‘probiotics’ (*I^2^* = 84%, *P*<0.001) and ‘prebiotics’ (*I^2^* = 90%, *P*<0.001) according to the types of microbial agents, indicating that the differences between the specific virus species might cause the final heterogeneity. Considering that there was no clear decrease in heterogeneity in the subgroup analyses by microbial agents’ types, we conducted a sensitivity analysis and discovered that the study performed by Maramatsu et al. was the main source of heterogeneity. We also evaluated the publication bias using Stata data analysis and determined a distinct publication bias based on Egger’s test (*P*<0.001).

**Table 3:** Subgroup and sensitivity analysis of viral load in preclinical studies

	Total number of studies	Total sample size		Subgroup analysis			Sensitivity analysis		
		Exp.	Ctr.	SMD (95% CI)	*I^2^*	*P*	SMD (95% CI)	*I^2^*	*P*
Effect estimates									
SEM	10	132	121	−1.14 (−1.47, −0.82)	22%	0.24	−1.05 (−1.33, −1.77)	0%	0.73
SD	19	461	275	−1.26 (−1.63, −0.88)	79%	<0.001	−1.16 (−1.51, −0.80)	75%	<0.001
Viral type									
H1N1	15	283	181	−1.54 (−1.98, −1.10)	73%	<0.001	−1.44 (−1.86, −1.01)	69%	<0.001
Others	4	208	104	−0.42 (−0.66, −0.18)	0%	0.53	-	-	-
Microbial agents									
Prebiotics	4	53	43	−2.39 (−4.16, −0.61)	90%	<0.001	−3.07 (−3.83, −2.30)	0%	0.4
Probiotics	15	438	242	−1.00 (−1.46, −0.54)	84%	<0.001	−0.86 (−1.25, −0.46)	79%	<0.001

Data were analyzed using a random-effects model. We analyzed the Effect estimates group for the included preclinical studies regarding viral load. We analyzed the viral type and microbial agent groups in the preclinical studies using SD as the effect estimate. Abbreviations: Ctr, control group; Exp, experimental group.

#### Cytokines

**Figure 4:**
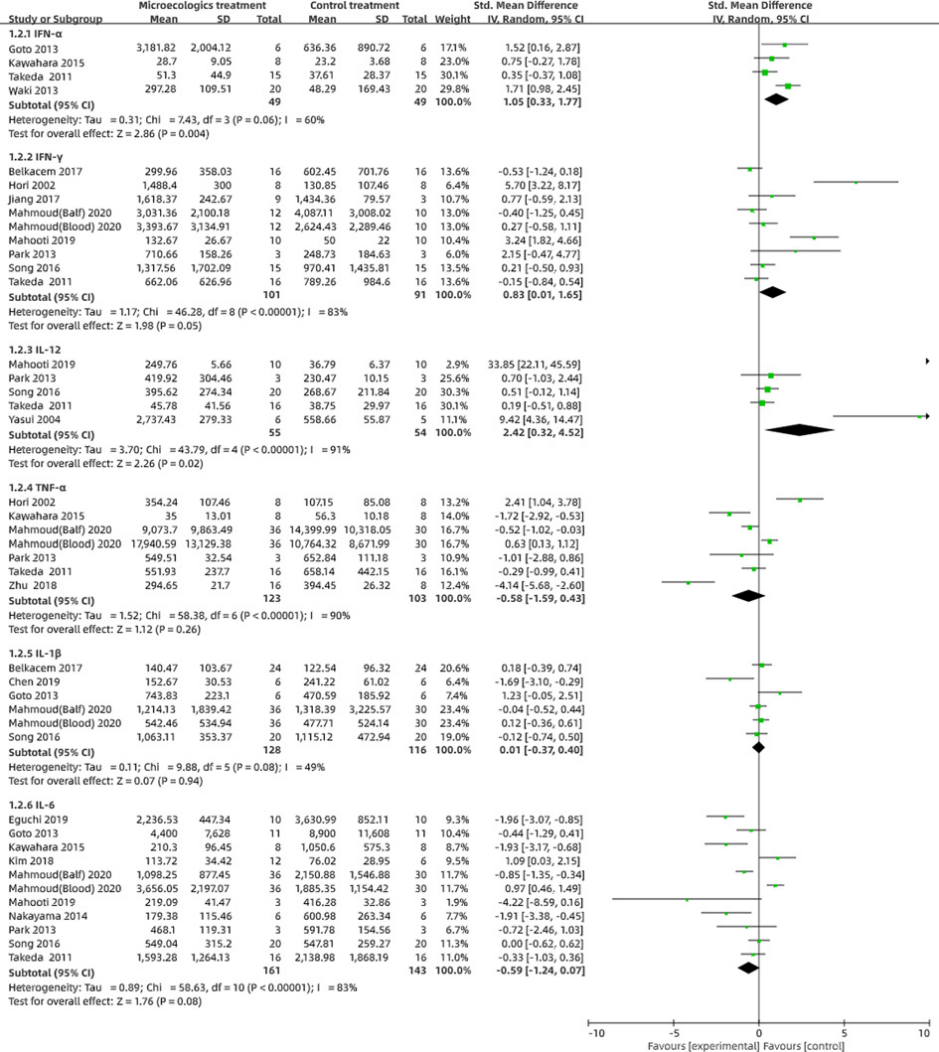
Evaluation of cytokines in preclinical studies We evaluated the IFN-α, IFN-γ, IL-12, TNF-α, IL-1β and IL-6 concentrations compared with the control group through a random-effects models using RevMan 5.3. The pooled data are expressed as SD with 95% CIs. *I^2^* and *P*-values represent the heterogeneity among the studies, while an *I^2^* value >50% and a *P*-value <0.1 indicate considerable heterogeneity. The 95% CI of the result intersecting with the solid vertical line represents no statistical significance.

We conducted forest plots to assess the changes in cytokines including interferon (IFN)-α, IFN-γ, tumor necrosis factor (TNF)-α, interleukin (IL)-1β, IL-12 and IL-6 levels in the studies (Figure 4). Based on the established readings, the consumption of probiotics or prebiotics increased the concentrations of IFN-α (SD: 1.05; 95% CI: 0.33–1.77; *P*=0.004), IFN-γ (SD: 0.83; 95% CI: 0.01–1.65; *P*=0.05), IL-12 (SD: 2.42; 95% CI: 0.32–4.52; *P*=0.02) and IL-1β (SD: 0.01; 95% CI: −0.37 to 0.40; *P*=0.94), with significant heterogeneity (*I^2^* = 60%, *P*=0.06; *I^2^* = 83%, *P*<0.001; *I^2^* = 91%, *P*<0.001 and *I^2^* = 49%, *P*=0.08, respectively) while it decreased the concentrations of TNF-α (SD: −0.58; 95% CI: −1.59 to 0.43; *P*=0.26) and IL-6 (SD: −0.59; 95% CI: −1.24 to 0.07; *P*=0.08), with significant heterogeneity (*I^2^* = 90%, *P*<0.001 and *I^2^* = 83%, *P*<0.001, respectively).

#### Adverse events

In most animal studies, the adverse events of probiotics and prebiotics are not recorded for lack of observable consequences. The reported aspiration pneumonia could be induced by either intranasally administrating high doses of live *L. rhamnosus* or the same dose of dead *L. rhamnosus* [22].

### Primary outcomes in clinical studies

#### Clinical symptoms

**Figure 5:**
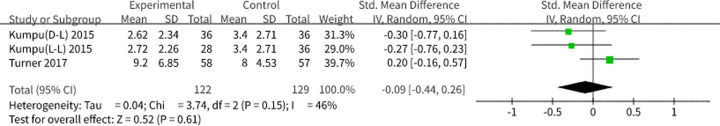
Symptom scores in the clinical studies The total symptom scores reported in the studies represent the symptom severity evaluations, and higher scores represent more severe symptoms. The pooled results are expressed as SD with 95% CI. *I^2^* and *P*-values represent the heterogeneity among the studies, while an *I^2^* value >50% and a *P*-value <0.1 indicate considerable heterogeneity. When the result intersects the invalid line, the result has no statistical significance. Abbreviations: D-L, dead *Lactobacillus*; L-L, live *Lactobacillus.*

Results from the pooled random-effects model analysis, showed a decrease in clinical symptom scores (SMD: −0.09; 95% CI: −0.44 to 0.26, *P*=0.61), with moderate heterogeneity (*I^2^* = 46%, *P*= 0.15) (Figure 5), implying a reduction in disease severity, although the result did not have a statistical significance because of small samples.

#### Infection rate

**Figure 6:**
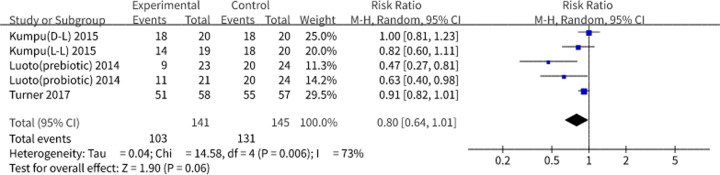
Viral infection rates in the clinical studies The pooled results are expressed as RR with 95% CI. *I^2^* and *P*-values represent the heterogeneity among the studies, while an *I^2^*value >50% and a *P*-value <0.1 indicate considerable heterogeneity. When the result intersects the invalid line, the result has no statistical significance. Abbreviations: D-L, dead *Lactobacillus*; L-L, live *Lactobacillus.*

Three studies that consisted of five subgroups including a highly heterogenetic subgroup focused on prebiotics were pooled for analysis using the random-effects model, As a consequence, an overall risk ratio (RR) and 95% CI of 0.80 and 0.64–1.01 (*P*=0.06) were shown in Figure 6. The results suggested that a decreased viral morbidity was due to the treatment of probiotics and prebiotics. If larger sample size were used for the analysis, the observed treatment effects could be statistically significant [20].

#### Adverse events

Among the studies were pooled for analysis, only Turner et al. reported gastrointestinal adverse events after treatment with probiotics, but no details were described [18].

#### Quality assessment

For clinical studies, all targets included for evaluation had low risk of selection bias and performance bias because they were randomized, double-blind, placebo-controlled trials. Most of the studies had low risk of reporting bias and other bias except for one study that had high risk of attrition bias [18] (Table 2). Quality evaluating in preclinical studies was detailed in Supplementary Table S1.

## Discussion

With the exploration of microbial agents, their properties that favor successful defense against respiratory virus infection have gaining a mass of interests. Olaimat et al. presented clinical fruits of the use of probiotic supplementation to prevent or treat respiratory tract infections [23] while Shinde et al. determined to identify evidence relating to potential mechanisms [24]. However, they just elaborated this subject qualitatively. Our study aimed to evaluate the effectiveness of on probiotics and prebiotics for viral pneumonia quantitatively, through the published data from 45 preclinical and 6 clinical studies. The probiotics included the live probiotics such as *L. rhamnosus* [25–28], *Bifidobacterium* [29,30] and a heat-inactivated one *Enterococcus faecalis* [13,31]. The prebiotics were commonly used such as inulin [32], polysaccharide [13,33–35] and oligosaccharide [36]. As a result, we found that both probiotics and prebiotics played a crucial role in treating viral pneumonia.

In viral pneumonia, inflammatory cascades could be commonly observed. Our results showed that probiotics or prebiotics helped to reduce the viral load leading to increase the overall survival through the up-regulation of the antiviral cytokines, IFN-α, IFN-γ, IL-1β and IL-12 and the down-regulation of the other cytokines IL-6 and TNF-α. The results from the cohort clinical studies on the influenza virus are generally consistent with those from preclinical studies. Patients treated with probiotics or prebiotics had lower disease severity and fewer infections. However, these findings warrant further studies to understand the effectiveness of probiotics and prebiotics in preventing viral pneumonia becuase of currently insufficient clinical studies and coherent indicators.

Interferon is a crucial antiviral factor that has a vital role in assessing host immunity. Unfortunately, there was no measure valid for the benefit of interferon in clinic yet. Our analysis revealed that all the probiotics or prebiotics tested could notably increase the interferon levels, consistent with a previous study that healthy athletes increased interferon secretion after a 1-month course of *L. acidophilus.* through a mechanism of engaging Toll-like receptors on the surface of antigen-presenting cells, which would, in turn, affect the subsequent cytokine secretion pattern [37,38], thereby indicating that probiotics and prebiotics work energetically by enhancing the host’s antiviral capabilities. Toll-like receptors (TLRs) play crucial roles in the innate immune system by recognizing pathogen-associated molecular patterns derived from various microbes. It has been reported that both neutrophil granulocytes and regulatory T cells put a halt to the reaction by releasing quantities of anti-inflammatory chemicals when an inflammatory response occurs within a capable immune system [14,39]. However, when the host is immunocompromised, effective responses break down. Accordingly, viral duplication that exacerbates tissue damages is attributed to a burst of inflammatory cytokines induced. Typically, the critical COVID-19 patients reported with higher plasma concentrations of IL-7, IL-8, IL-9, basic fibroblast growth factor, granulocyte colony-stimulating factor and granulocyte-macrophage colony-stimulating factor were in high fatality [6].

A well-documented postbiotic is called short-chain fatty acids (SCFAs) is that are produced by bacterial fermentation of indigestible fibers. The most abundant SCFAs refer to propionate, acetate and butyrate. SCFAs act on G protein-coupled receptors (GPRs) 41 and GPR43, [40,41] or as histone deacetylase inhibitors [42] to down-regulate proinflammatory chemokine and combat cytokine cascades. Our findings demonstrated that immune cells took advantage of bacterial metabolites to enhance antiviral response. The mucosal immune system, specifically the lymphoid tissues on the mucosal surface, was also involved in modulating anti-virus immunity [43,44]. Microfold cells could absorb bacterial metabolites into the circulation, with a bond of mucosal immune system where they would stimulate immune cells and rapidly recruit them [45].

IL-12 and IL-6 are representative activators to Th1 and Th2, respectively. In severe influenza infections, there was a marked Th polarization shift from Th2 to Th1 [46]. However, during probiotics or prebiotics treatment, this inflammation-oriented polarization could be reversed through up-regulation of IL-12 and down-regulation of IL-6. Furthermore, Chen et al. and other researchers discovered that production of IL-10 from Treg cells was increased [13,25]. IL-10 functions to limit the host immune response to pathogens, thereby preventing damage to the host and maintaining normal tissue homeostasis. Thus, we can speculate that probiotics and prebiotics could recruit Treg cells and up-regulate IL-10 concentrations to achieve an antiviral effect by preventing immoderate inflammatory responses through inhibiting production of the inflammatory cytokines, TNF-α and IL-6.

Concerning secondary infections incurred by enterogenous endotoxemia during severe viral pneumonia, the gut microbiota could notably defend it. The gut mucosal barrier is composed of mucus, symbiotic flora, tight junctions between intestinal epithelial cells and mucosal immune cells, making it difficult for opportunistic infections to take root. Once the virus fiercely strikes at the respiratory tissues, it would possibly bring about systematic hypoxia, where the intestinal epithelial cells would dysfunction and act to weaken the mucosal barrier [14]. In addition, the misuse or overuse of broad-spectrum antibiotics could invariably result in dysbiosis, blemishing intestinal permeability and endamaging gut mucosal barrier during antiviral treatment [47].

Probiotic supplements were considered to be an optimal approach to restore the gut mucosal barrier function in viral pneumonia. Probiotics can bind to Toll-like receptor-4, whose population could increase with the help of inactivated *L. salivarius* and fructo-oligosaccharides [48], thereby competing against harmful bacteria. In addition, probiotics and its metabolic profiles including bacteriocin, hydrogen peroxide, antimicrobial peptides and defensin, help to modulate the local immunity and drive enterocyte and goblet cells to secret mucus as a consequence to strengthen the mucosal barrier at length [49,50].

For COVID-19 patients, SARS-CoV-2 binds its spike proteins to angiotensin‐converting enzyme 2 (ACE2). ACE2 is highly expressed in the bronchi and gastrointestinal tract to facilitate to viral invading and replication [51,52]. Since the invasive bindings to ACE2, ACE2 located in the gut might not function effectively, potentially altering the symbiotic flora and undermining the intestinal barrier, leading to patients prone to secondary infections. A published study reported that treatment of an irritant, compared with wildtype littermates, caused gut microbiota alteration to promote profoundly inflammatory reaction in ACE2 mutation mice, which could be directly regulated by microbial agents [53]. Encouragingly, probiotics and prebiotics treatment has been incorporated as adjuvant therapy for critical patients to prevent secondary infections in the fourth Trial Edition of COVID-19 Diagnosis and Treatment Plan by the National Health Commission of China, [54].

In summary, our study suggested that probiotics and prebiotics could be an inspiration for healthcare givers when treating viral pneumonia. This therapy could limit inflammatory responses, stimulate both innate and adaptive immune cells to defend against the viral attacks and preventing secondary infections. Our findings implied a promising target and encourage probiotics or prebiotics to be incorporated into regular treatments for patients infected with respiratory virus, particularly for the patients with severe viral pneumonia.

## Limitations

The present study has several limitations. Firstly, most of the clinical studies related to the topic were not included because they focused generally on the respiratory tract infections but not particularly on respiratory viral infections. Thus, only a small number of clinical studies was eligible for our analysis, thereby influencing the extrapolation of outcomes. Secondly, considering the differences in experimental designs, we did not conduct a direct comparison of the merits of individual microecological agents tested. We also did not confirm optimal dosage, dosage form and duration, which need further investigation. Thirdly, despite remarkable functions showed in applying probiotics and prebiotics to treat viral pneumonia, the effects only limit to a certain amount of bacteria species and their products. Therefore, it is appropriate to specify the individual probiotics or prebiotics with more explorations.

## Supplementary Material

Supplementary Table S1

## Data Availability

All data generated or analyzed during the present study are included in this published article.

## Abbreviations

ACE2: angiotensin-converting enzyme 2
CAP: community-acquired pneumonia
CI: confidence interval
COVID-19: novel coronavirus pneumonia
GPR: G protein-coupled receptor
HR: hazard ratio
IFN: interferon
IL: interleukin
RR: risk ratio
RSV: respiratory syncytial virus
SARS: severe acute respiratory syndrome
SCFA: short-chain fatty acid
SD: standard deviation
TNF: tumor necrosis factor
